# Lemons, or Squeezed for Resources? Information Symmetry and Asymmetric Resources in Biotechnology

**DOI:** 10.3389/fphar.2017.00338

**Published:** 2017-06-02

**Authors:** Arthur Neuberger, Nektarios Oraiopoulos, Donald L. Drakeman

**Affiliations:** ^1^Department of Pharmacology, University of CambridgeCambridge, United Kingdom; ^2^Cambridge Judge Business School, University of CambridgeCambridge, United Kingdom

**Keywords:** in-licensing, drug development, information asymmetry, clinical trials design and analysis, pharmaceutical industry

## Abstract

Thousands of biotech companies are developing promising products, but have insufficient resources to complete the clinical testing process, while large, well-funded companies have increasingly focused on the need to access external innovation. As a result, licensing deals are an essential and growing part of this industry. Yet, casting a shadow over the licensing market is the classic Lemons Problem: Does asymmetrical information put licensees at a severe disadvantage, leading to a market dominated by inferior opportunities, with the best products retained for internal development? Our analysis of clinical stage products developed over three decades shows that there is no Lemons Problem. We discuss the results of this first apples-to-apples analysis of the biomedical licensing market, and suggest reasons why the Lemons Problem does not exist where it might be most expected—in a high technology, knowledge-based industry.

## Introduction

Multi-billion dollar investments and high failure rates are “business as usual” for the biopharmaceutical industry (DiMasi et al., 2016). With thousands of development-stage companies, most of which have limited access to the resources required to develop a new drug all the way through the FDA approval process, licensing deals have always been an integral part of this industry. In recent years, there has been an even greater emphasis on investments by large companies in external innovation (M&As, joint ventures, in-licensed drugs), with 2015 being an all-time record year for the industry (PriceWaterhouseCoopers, 2009). Yet, casting a shadow over this lively licensing market is the classic Lemons Problem identified by Nobel laureate George Akerlof: Will the licensors' superior knowledge about the products put licensees at a severe disadvantage, leading to a market dominated by inferior opportunities, with the best products retained for internal development?

Our analysis of all clinical stage products developed over three decades by the most prominent biotechnology companies shows that there is no Lemons Problem in biopharmaceutical licensing. In this article, we will discuss the results of this first apples-to-apples analysis of the biomedical licensing market, and suggest reasons why the Lemons Problem, a phenomenon taught to every first-year MBA student, does not exist where it might be most expected—in a high technology, knowledge-based industry.

## Background

The economic, finance and strategic management literature has often identified the information asymmetry that can lead to a Lemons Problem as an important element of transactions involving licensing and technology transfer in high technology industries (Gallini and Wright, 1990; Wuyts and Dutta, 2008), including the biotech industry (Lerner and Merges, 1998; Antelo, 2003; Audretsch and Feldman, 2003; Rothaermel and Deeds, 2004; Pisano, 2006, unpublished manuscript; Mason et al., 2008). The central hypothesis has been that the company originating the product has superior information compared to the in-licensor, and as such it would only seek to out-license drugs of inferior quality while keeping the good ones for internal development.

Empirical studies of various types of inter-firm transactions—including licenses, partnerships, and other alliances—have produced mixed results on this issue. Pisano observed a Lemons Problem in a study of 260 biotechnology projects, but the analysis assumed that multiple indications are the equivalent of multiple products, which is not representative of the biotech licensing market, in which indication-splitting is rare (Pisano, unpublished manuscript). Arora et al. (2009), who found no evidence of a Lemons Problem, focused only on preclinical stage products, and again treated separate indications as if they were separate products. A third recent analysis of products in-licensed by the 50 largest pharmaceutical companies suggests the opposite of a Lemons Problem. This study by DiMasi et al. (2010) seems to support the conclusion that drugs in-licensed by the 50 largest pharmaceutical companies (ranked by sales) have higher success rates than those developed in-house, although because the timing of in-licensing is not specified, this observation could be driven by comparing later stage in-licensed products with in-house products that were selected for development on the basis of only preclinical data.

Whether a Lemons Problem exists in biotech licensing, especially for clinical stage products, thus remains an open and important question. Economic theory predicts that it should, but the studies to date have been inconclusive. Perhaps the licensor's knowledge of the product and the underlying technology leads to information asymmetry and a Lemons Problem. Yet, considering the high failure rates and the multiple “unknown unknowns” involved in the drug development process (Hay et al., 2014), it is far from certain that the out-licensing party will necessarily have any better knowledge than the licensee of the drug's ultimate potential to treat a specific disease safely and effectively enough to receive FDA approval.

Our study directly addresses this issue: is there a Lemons Problem in biotech licensing transactions, as evidenced by the success rates of internally retained products compared to those that have been out-licensed. In designing the analysis, our goal has been to make as direct a comparison as possible. One key characteristic of prior studies is that they do not control for when in the development process each of the licensed-in drugs was acquired. Moreover, we exclude any marketing or co-development agreements, so that our final sample consists of only pure licensing agreements. Finally, we have included in the dataset only transactions in which biotech companies were both licensee and licensor.

## Data structure and methods

In order to assess the existence of a Lemons Problem in biotech licensing transactions, we assembled a hand-annotated sub-data set on the basis of Thomson Reuters' Recap BioPortfolio Index (RBI) of 170 biotech firms. According to Recap IQ, RBI companies are chosen based on their size, breadth of clinical programs, revenue (if relevant), and scientific innovation to serve as a representative benchmark data set for the industry. Importantly, “these companies also tend to focus on new, untested technologies (recombinant DNA, monoclonal antibodies, and novel molecular targets) and unmet medical needs (rather than developing “me-too” or second-generation products) to a greater degree than “traditional” pharmaceutical companies” (Recap, 2016). These are the types of products where the novelty of the technology would seem to create the strongest possibility of information asymmetry.

This annotated set of data enabled us to investigate the full clinical development history for the total population of drugs that were launched by these 170 biotech companies into clinical development between 1980 and 2012. Pharmaceutical companies, defined as any drug development company founded before 1975, are excluded in this data set. From this total population, we extracted 196 in-licensed and 634 in-house originated drug development cases with a determined outcome, i.e., those cases for which all initiated first and secondary indication trials have either been terminated or have led to a market launch of the drug. Market launch success versus failure was determined for each individual drug development case. A “success” case was defined as any market launch or approval of the drug for any indication. Multiple launches of the same drug for various indications were still treated as a single success case. Correspondingly, only if all first and secondary indications were terminated was the drug development project classified as a “failure” case. Drugs with uncompleted and ongoing clinical trials were not considered in this study. The 196 in-licensing cases were re-categorized into sub-populations according to the clinical stage at which the compound was in-licensed (see Table 1).

**Table 1:** Number of in-licensed and in-house developed projects in phases I-III in utilized biotech data set.

**Clinical trial**	**In-house**	**In-licensed**
Phase I	634	48
Phase II	439	52
Phase III	190	17

Using this data set, we compared the success rates of: (i) phase I in-house originated drugs vs. drugs in-licensed in phase I; (ii) phase II in-house originated drugs vs. drugs in-licensed in phase II. Phase III drugs were excluded from our analysis due to the small sample size of in-licensed drugs (*n* = 17) (see Table 1).

## Findings and discussion

**Figure 1:**
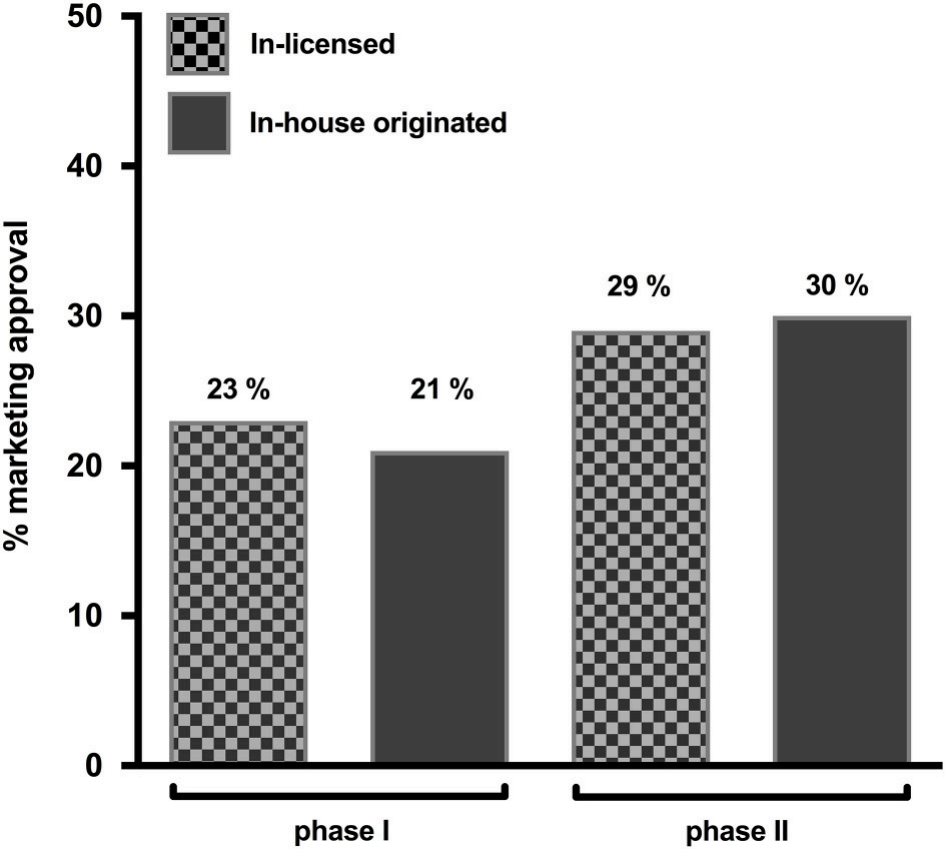
Marketing approval success rate comparison between in-licensed and in-house originated drugs in phases I and II.

Our main finding (depicted in Figure 1) is that, for the biotech industry, in-licensed and in-house products had statistically comparable success rates. Specifically, we find that phase I licensed-in drugs have a success rate of 23%, which is comparable to the 21% success rate of in-house developed drugs. Similarly, phase II licensed-in drugs have a success rate of 29%, which is not statistically different from the 30% of in-house developed drugs. Taken together, these results suggest that there appears to be no Lemons Problem in the biotechnology industry.

In 2008, Myriad Genetics licensed to H. Lundbeck the European rights to Flurizan, a product that had successfully completed Phase II trials and was then in Phase III trials. Flurizan was being developed to treat Alzheimer's disease, and Lundbeck was an international pharmaceutical company that specialized in the development and commercialization of products for central nervous system diseases such as Alzheimer's. Lundbeck paid Myriad $100 million upon signing the agreement and agreed to pay up to $250 million in connection with regulatory approvals, plus commercial milestones and royalties. Six weeks later, Flurizan failed to achieve satisfactory Phase III results and was abandoned.

While that example looks like strong evidence of a Lemons Problem, the industry can provide plenty of counter-examples. In 1995, biotech company Idec Pharmaceuticals entered into a licensing transaction with Genentech. The primary product involved in the transaction was a monoclonal antibody that had completed Phase II clinical trials. Genentech acquired a >50% commercial interest in the product, plus an option to acquire commercial interests in two additional products, for an upfront payment of $9 million ($5 million of which was in the form of a purchase of preferred stock), a commitment to purchase $17.5 million of Idec equity, and $30.5 million in milestone and option payments. The product, now called Rituxan, was approved 2 years later, and has become one of the biopharmaceutical industry's most successful blockbusters. Genentech's total investment of less than $60 million rapidly returned hundreds of millions of dollars of annual profits from the sales of Rituxan, plus a substantial return on the equity investment. By 2008, worldwide sales of Rituxan reached approximately $5 billion.

Seen together, these two examples emphasize the impressive number of unknowns in the drug development process, which is likely to lead to a lack-of-information symmetry shared by licensor and licensee alike. In these transactions, the licensor and licensee may have different opinions about the likelihood of success, the time, and cost of further development, the strength of patent protection, the size of the market and other common drug development variables, but it is not clear that there is knowledge available to the licensor that puts it in the position of someone trying to get rid of a lemon, as in Akerlof's famous example of a used car seller—i.e., possessing otherwise hidden technical knowledge that provides the seller with a distinct advantage over the buyer in assessing the value of the item being sold (Akerlof, 1970). Unlike Akerlof's owner of a perfectly running used car, who has no reason to sell, especially into a market that discounts its value for defects that are hidden to the buyers, a biotech company may license even its “crown jewels” to another company if it does not otherwise believe that it will have access to the financial resources to survive and to develop the product on its own. By analogy, Akerlof's car owner might well sell a perfect car, even in a discounted market, if he cannot afford to put gas in it. Meanwhile the party with plenty of cash for gas, that is, a larger company with abundant resources and an inadequate internal pipeline, has an urgent need for the car. This need of licensors for resources, and of licensees for products to fill the pipeline, is a strong enough motivation for both companies to pursue licensing transactions with considerable enthusiasm.

The licensing literature has often described information asymmetry in biotech licensing transactions largely because it was expected to be there. That it is not seen in our analysis indicates that the parties to licensing transactions are able to achieve an adequately symmetrical understanding of the product and its potential to avoid a Lemons Problem. The explanation may differ between research and clinical stage transactions. At the preclinical stage, the impressively low rates of success mean that neither party has access to information that would enable it to predict the ultimate outcome. In that respect, the preclinical licensing business is less like a market for lemons than for buying lottery tickets—that is, almost all drug development projects will be the equivalent of Akerlof's totally defective car—or a losing lottery ticket—with no ability of either party to predict with confidence which product might turn out to be the one-in-one-hundred opportunity that will be a successful drug. Once the product moves into clinical testing, information symmetry continues, but probably for different reasons. As predictability gradually increases through the clinical development process, the FDA's requirements for rigorous data collection ensure that licensees can, with diligent effort, become as knowledgeable as licensors. Even then, drug development remains remarkably unpredictable, but licensors and licensees share that problem equally, thanks to the regulatory environment and a due diligence process designed to avoid any potential lemons. In short, because the Lemons Problem exists in theory, it does not appear to exist in practice.

Even though the Lemons Problem may not exist, it still makes sense to teach MBAs about it. The concerns raised by Akerlof's hypothesis have created a licensing environment in which the licensees' due diligence process is extremely detailed and quite lengthy. It is not unusual for critical experiments to be repeated in a different laboratory, for lab notebooks to be scrutinized by scientific experts, and for individual patient records to be analyzed by health professionals, all to ensure that the information provided by the licensor is complete and correct. Moreover, the regulatory process required for human clinical testing necessarily creates a large body of information that is designed for the FDA's use, but which is equally valuable for potential licensees in creating a level and symmetrical playing field for the two parties to consider a transaction. In short, by the time the due diligence process is complete, a knowledgeable licensee should have as much information about the licensing candidate as it does for its own products at the same stage of development, hence our finding that the success rates of in-licensed and in-house products are comparable.

## Author contributions

All authors listed, have made substantial, direct and intellectual contribution to the work, and approved it for publication.

### Conflict of interest statement

The author DD has financial interests in numerous biotechnology companies. The other authors declare that the research was conducted in the absence of any commercial or financial relationships that could be construed as a potential conflict of interest.
